# Hemodynamic Patterns in Septic Cardiomyopathy: Insights From Echocardiographic Parameters and Biomarker Analysis

**DOI:** 10.7759/cureus.91283

**Published:** 2025-08-30

**Authors:** Rabia Jaffar, Ahmed Jamal Chaudhary, Marium Nadeem Khan, Hamza Wakil, Muhammad Waseem Hussain, Mohammad Ibrahim Rasool, Taha Nasim, Muhammad Haris Khan, Ali Raza

**Affiliations:** 1 Internal Medicine, Tunbridge Wells Hospital, Tunbridge Wells, GBR; 2 Internal Medicine, Liaquat University of Medical and Health Sciences, Jamshoro, PAK; 3 Internal Medicine, Michigan State University, Detroit, USA; 4 Medicine, Shifa College of Medicine, Islamabad, PAK; 5 Gastroenterology and Hepatology, Royal Alexandra Hospital, NHS Greater Glasgow and Clyde, Paisley, GBR; 6 Cardiology, Khyber Teaching Hospital, Peshawar, PAK; 7 Pediatrics, Naseer Teaching Hospital, Peshawar, PAK; 8 Pediatrics, Kabir Medical College, Peshawar, PAK; 9 Emergency, Zubaida Medical Center/Sindh Medical College, Karachi, PAK; 10 Accident and Emergency, Medical Teaching Institution (MTI) District Headquarter (DHQ) Teaching Hospital, Dera Ismail Khan, PAK; 11 Cardiology, Khyber Teaching Hospital, Medical Teaching Institution (MTI), Peshawar, PAK

**Keywords:** echocardiography, global longitudinal strain, icu outcomes, nt-probnp, sepsis, septic cardiomyopathy, troponin

## Abstract

Introduction

Septic cardiomyopathy (SCM) refers to an acute, reversible impairment of cardiac performance. This study aimed to evaluate hemodynamic patterns in SCM using echocardiographic parameters and cardiac biomarkers in critically ill patients.

Materials and methods

A 12-month prospective observational study was conducted at a single tertiary care center in Peshawar, Pakistan. Adult ICU patients with sepsis or septic shock underwent transthoracic echocardiography (TTE) within 48 hours of admission, with a repeat study on day 5 or earlier if clinically indicated. Left ventricular (LV) and right ventricular (RV) systolic and diastolic functions were assessed, including global longitudinal strain (GLS) and RV free-wall strain. High-sensitivity troponin I (hs-TnI) and N-terminal pro-B-type natriuretic peptide (NT-proBNP) were measured on days 1 and 3. Associations between cardiac dysfunction and in-hospital mortality were analyzed using multivariable logistic regression after adjusting for age, sex, Acute Physiology and Chronic Health Evaluation II (APACHE II) score, and vasopressor use.

Results

Among 122 patients, the prevalence of SCM, defined by LV GLS > −17%, was high, with concurrent RV dysfunction observed in a significant proportion. Elevated hs-TnI and NT-proBNP levels correlated with impaired GLS and adverse outcomes. After multivariable adjustment, GLS impairment and elevated NT-proBNP remained independently associated with in-hospital mortality.

Conclusions

In this South Asian cohort with sepsis or septic shock, combined strain-based echocardiography and biomarker profiling revealed distinct patterns of myocardial dysfunction associated with early mortality. While these findings highlight the prognostic utility of GLS and NT-proBNP, they should be interpreted as associations rather than causal effects. Larger multicenter studies with extended follow-up and incorporation of additional biomarkers and advanced imaging are needed to validate these observations and clarify underlying pathophysiological mechanisms.

## Introduction

Sepsis remains a leading cause of admission to intensive care units (ICUs) worldwide, accounting for an estimated 19 million cases annually and mortality rates that still hover between 17% and 33% despite advances in antimicrobial therapy and organ support strategies [1]. The Surviving Sepsis Campaign highlights that early identification and hemodynamic optimization are pivotal, yet many survivors sustain long-term cardiovascular sequelae, underscoring the need to understand sepsis-related myocardial dysfunction more deeply [2].

Septic cardiomyopathy (SCM), as defined within the framework of Sepsis-3 criteria, refers to an acute, reversible impairment of myocardial function that arises in the setting of sepsis or septic shock and is not explained by pre-existing structural heart disease or acute coronary syndrome. Reported in up to 44% of septic patients, SCM encompasses a spectrum ranging from isolated diastolic impairment to biventricular systolic failure, and its presence has been associated with a two- to three-fold increase in short-term mortality [3]. Although its pathophysiology involves a complex interplay of inflammatory cytokines, mitochondrial dysfunction, nitric-oxide-mediated myocardial depression, and altered β-adrenergic signaling, the absence of universally accepted diagnostic criteria has led to heterogeneity across studies [4].

Hemodynamically, sepsis often evolves from an early hyperdynamic state characterized by high cardiac output and low systemic vascular resistance to a later hypodynamic phase with depressed contractility and poor perfusion. Contemporary echocardiography has enabled bedside delineation of these phases: conventional left ventricular ejection fraction (LVEF) may remain “normal” in hyperdynamic sepsis, whereas speckle-tracking-derived global longitudinal strain (GLS) unmasks subclinical systolic failure; similarly, tissue Doppler indices and E/e′ ratios provide insight into diastolic dysfunction that traditional preload markers overlook [5]. Right ventricular (RV) involvement is increasingly recognized, with recent series demonstrating RV dysfunction in nearly half of septic shock patients and an independent three-fold increase in 28-day mortality [6].

Serum biomarkers complement imaging by reflecting cardiomyocyte injury and neurohormonal stress [7]. Elevated cardiac troponin concentrations correlate with disease severity and prolonged mechanical ventilation days, while natriuretic peptides (B-type natriuretic peptide [BNP] or N-terminal pro-B-type natriuretic peptide [NT‑proBNP]) mirror ventricular stretch but are confounded by sepsis-related renal impairment [8]. Emerging mediators such as high mobility group box 1 protein, soluble suppression of tumorigenicity 2 (sST2), and growth differentiation factor 15 offer mechanistic clues but lack bedside availability. Integrating serial biomarker trends with dynamic echocardiography could refine risk stratification and guide tailored fluid, vasopressor, and inotrope therapy, yet prospective evidence remains sparse [9].

A definitive hemodynamic phenotype of SCM that integrates comprehensive echocardiographic metrics (including left ventricular [LV] and RV strain) with serial cardiac biomarker profiling has not been established in South Asian adult populations. Therefore, the primary objective of this study was to characterize SCM according to Sepsis-3 definitions by assessing LV and RV function using advanced echocardiographic parameters (LVEF, GLS, tricuspid annular plane systolic excursion [TAPSE], fractional area change [FAC], RV strain) within 72 hours of admission. Secondary objectives were to (i) evaluate correlations between echocardiographic parameters and cardiac biomarkers (high-sensitivity troponin I [hs-TnI] and NT-proBNP), (ii) determine the association of SCM with illness severity scores (Acute Physiology and Chronic Health Evaluation II [APACHE II] and Sequential Organ Failure Assessment [SOFA]), and (iii) assess the prognostic significance of SCM for in-hospital mortality, vasopressor requirement, and prolonged mechanical ventilation.

## Materials and methods

Study design and setting

This prospective observational study was conducted at Khyber Teaching Hospital MTI (Medical Teaching Institution), Peshawar, Pakistan. The study spanned a period of 12 months (June 19, 2023, to June 19, 2024).

Sample size calculation

The sample size was calculated using OpenEpi version 3.01. Based on a previous study that reported the prevalence of SCM at approximately 40% in critically ill septic patients [10] and using a 95% confidence level with a 9% margin of error, the minimum sample size was estimated to be 106. To account for possible data loss or incomplete follow-up, a total of 122 patients were enrolled.

Inclusion and exclusion criteria

Patients were included if they were aged 18 years or older, diagnosed with sepsis or septic shock according to Sepsis-3 definitions, admitted to the ICU within 24 hours of sepsis onset, and underwent transthoracic echocardiography (TTE) within the first 48 hours of admission. Patients were excluded if they had pre-existing structural heart disease, a documented LVEF < 50% prior to admission, recent acute coronary syndrome or myocardial infarction (within six weeks), chronic kidney disease requiring dialysis, or an inadequate echocardiographic window.

Data collection

A total of 122 patients meeting the inclusion criteria were enrolled consecutively. Demographic data, comorbidities, source of infection, and APACHE II and SOFA scores were recorded at admission. Laboratory investigations included complete blood count, renal and liver function tests, and inflammatory markers. Cardiac biomarkers - hs-TnI and NT-proBNP - were measured on days 1 and 3 using standard laboratory protocols.

Echocardiographic assessment

Bedside TTE was performed by trained intensivists using a GE Vivid S70 ultrasound system (GE HealthCare, Chicago, IL). GLS and RV strain were analyzed offline with EchoPAC software (GE Healthcare) according to ASE/EACVI recommendations, with frame rates of 60-90 fps and averaging from three apical views. To enhance reproducibility, two independent readers performed analyses, and inter-observer variability was assessed in a random subset of 20 studies. Assessments were conducted within 48 hours of ICU admission and repeated on day 5 or earlier if clinical deterioration occurred.

LV systolic function was evaluated using Simpson’s biplane LVEF and GLS. Diastolic function was assessed using E/A ratio, E/e′, and deceleration time, with grading performed according to ASE/EACVI criteria. RV function was evaluated by TAPSE, RV FAC, and RV free wall longitudinal strain. RV dysfunction was defined as TAPSE < 17 mm, FAC < 35%, or RV free wall strain > −20% [11].

Definitions

In line with Sepsis-3, SCM was defined as new-onset LV or RV systolic or diastolic dysfunction during sepsis in the absence of pre-existing cardiac pathology. SCM was operationalized as LV GLS > −17% on echocardiograms performed within 72 hours of admission and/or RV dysfunction by the aforementioned criteria [12]. Elevated hs-TnI (>99th percentile of normal) and NT-proBNP levels were interpreted in relation to myocardial injury and ventricular strain.

Statistical analysis

The primary endpoint was in-hospital mortality. Secondary outcomes included vasopressor requirement >72 hours, prolonged mechanical ventilation (>5 days), and ICU length of stay. Data were entered and analyzed using IBM SPSS Statistics Version 26 (IBM Corp., Armonk, NY). Continuous variables were expressed as mean ± standard deviation or median (IQR), depending on distribution. Categorical variables were expressed as frequencies and percentages. Between-group comparisons (SCM vs. no SCM) were performed using Student’s t-test or Mann-Whitney U test for continuous variables and chi-square or Fisher’s exact test for categorical variables. Pearson’s or Spearman’s correlation was used to explore associations between echocardiographic parameters and biomarkers. To account for confounding, multivariable logistic regression was performed including age, sex, APACHE II score, vasopressor use, and renal function (serum creatinine/estimated glomerular filtration rate [eGFR]). A p-value of <0.05 was considered statistically significant.

Treatment and follow-up

All patients received standard care according to the Surviving Sepsis Campaign guidelines, including timely antimicrobial therapy, source control, fluid resuscitation, and vasopressor support as indicated. No specific heart failure-directed therapies (e.g., beta-blockers, angiotensin-converting enzyme inhibitors) were initiated beyond sepsis management. Patients were followed during their ICU stay until discharge or death; no post-discharge follow-up was undertaken.

Ethical considerations

Ethical approval was obtained from the Institutional Research and Ethical Review Board (IREB) of Khyber Teaching Hospital MTI (719/DC/KMC) prior to the commencement of the study, and informed consent was obtained from patients’ legal representatives.

## Results

Among the 122 enrolled patients, 57 (46.7%) were diagnosed with SCM and 65 (53.3%) had no SCM. Patients with SCM had significantly higher illness severity scores, with a mean APACHE II score of 24.4 ± 6.3 compared to 21.1 ± 5.8 in the non-SCM group, with a mean SOFA score of 10.6 ± 2.9 versus 8.1 ± 2.8, respectively. The mean age was slightly higher in the SCM group (60.1 ± 13.7 years) compared to those without SCM (57.6 ± 14.6 years), though this was not statistically significant (t = 1.11, p = 0.271). Gender distribution was comparable between groups, with 34 (59.6%) males in the SCM group and 37 (56.9%) in the non-SCM group (χ² = 0.09, p = 0.763). Pulmonary infection was the most common source in both groups, occurring in 23 (40.4%) patients with SCM and 23 (35.3%) patients without SCM. Notably, in-hospital mortality was significantly higher in patients with SCM compared to those without SCM, at 29 (50.9%) versus 14 (21.5%), respectively, indicating a strong association between SCM and adverse outcomes. Baseline demographic and clinical characteristics are shown in Table 1. On multivariable logistic regression, higher APACHE II score (adjusted OR 1.08, 95% CI 1.02-1.15, p = 0.009) and higher SOFA score (adjusted OR 1.12, 95% CI 1.03-1.22, p = 0.004) were independently associated with SCM. In-hospital mortality remained significantly associated with SCM after adjustment (adjusted OR 2.45, 95% CI 1.11-5.38, p = 0.026). Age, sex, and pulmonary source of infection were not independent predictors.

**Table 1:** Baseline demographic and clinical characteristics *Adjusted for age, sex, APACHE II score, and vasopressor use. **Significant at p < 0.05. SCM, septic cardiomyopathy; APACHE II, Acute Physiology and Chronic Health Evaluation II; SOFA, Sequential Organ Failure Assessment

Variable	SCM (n=57)	No SCM (n=65)	Test (t/χ²)	p-Value (univariate)	Adjusted OR (95% CI)*	p-Value (multivariable)
Age (years), mean ± SD	60.1 ± 13.7	57.6 ± 14.6	t = 1.11	0.271	1.02 (0.99–1.05)	0.18
Male gender, n (%)	34 (59.6%)	37 (56.9%)	χ² = 0.09	0.763	1.12 (0.55–2.30)	0.75
Pulmonary source, n (%)	23 (40.4%)	23 (35.3%)	χ² = 0.38	0.566	1.20 (0.56–2.57)	0.64
APACHE II score, mean ± SD	24.4 ± 6.3	21.1 ± 5.8	t = 3.03	0.004	1.08 (1.02–1.15)	0.009**
SOFA score, mean ± SD	10.6 ± 2.9	8.1 ± 2.8	t = 4.69	<0.001	1.12 (1.03–1.22)	0.004**
In-hospital mortality, n (%)	29 (50.9%)	14 (21.5%)	χ² = 11.53	<0.001	2.45 (1.11–5.38)	0.026**

Cardiac biomarker levels were markedly elevated in patients with SCM. The mean hs-TnI on day 1 was significantly higher in the SCM group (158.2 ± 78.4 ng/L) than in the non-SCM group (86.7 ± 51.2 ng/L; t = 6.13, p < 0.001). This trend persisted on day 3, with hs-TnI still elevated in SCM patients (112.5 ± 60.3 ng/L vs. 65.1 ± 44.9 ng/L; t = 4.81, p < 0.001). Similarly, NT-proBNP levels on both day 1 and day 3 were significantly higher in the SCM group (day 1: t = 6.59, day 3: t = 5.53, both p < 0.001). These elevations reflect ongoing myocardial stress and injury. These data support the role of these biomarkers in the early detection and monitoring of SCM (Table 2).

**Table 2:** Cardiac biomarkers in patients with and without SCM *Significant at p < 0.05. SCM, septic cardiomyopathy; hs-TnI, high-sensitivity troponin I; NT-proBNP, N-terminal pro–B-type natriuretic peptide

Biomarker	SCM (n=57)	No SCM (n=65)	t-Value	p-Value
hs-TnI day 1 (ng/L)	158.2 ± 78.4	86.7 ± 51.2	6.13	<0.001*
hs-TnI day 3 (ng/L)	112.5 ± 60.3	65.1 ± 44.9	4.81	<0.001*
NT-proBNP day 1 (pg/mL)	4990.8 ± 1840.2	2,981.4 ± 1,557.3	6.59	<0.001*
NT-proBNP day 3 (pg/mL)	3617.2 ± 1631.6	2,114.6 ± 1,324.5	5.53	<0.001*

Echocardiographic assessment demonstrated significantly impaired cardiac function in patients with SCM. The mean LVEF was markedly reduced in the SCM group at 44.8 ± 7.5% compared to 56.2 ± 6.1% in the non-SCM group (t = 9.00, p < 0.001). Similarly, GLS was significantly impaired in SCM patients (−13.2 ± 2.9%) versus those without SCM (−17.5 ± 2.6%; t = 8.18, p < 0.001), highlighting early subclinical myocardial dysfunction. Diastolic dysfunction was more pronounced in the SCM group, as reflected by a higher mean E/e′ ratio of 17.1 ± 4.2 compared to 12.5 ± 3.1 (t = 6.75, p < 0.001).

RV systolic function was also compromised, with lower TAPSE values in the SCM group (15.2 ± 2.7 mm vs. 18.7 ± 3.0 mm; t = 6.54, p < 0.001). RV dysfunction was defined as TAPSE < 17 mm, FAC < 35%, or RV free wall strain > −20%. Using this definition, RV dysfunction was significantly more common in patients with SCM, observed in 22 (38.6%) individuals versus only 7 (10.8%) in the non-SCM group (χ² = 11.87, p < 0.001). These findings suggest that SCM involves both LV and RV impairment and that conventional LVEF measurements may underestimate the extent of cardiac dysfunction (Table 3).

**Table 3:** Echocardiographic parameters in both groups SCM, septic cardiomyopathy; LVEF, left ventricular ejection fraction; GLS, global longitudinal strain; E/e′, ratio of early mitral inflow velocity to early diastolic mitral annular velocity; TAPSE, tricuspid annular plane systolic excursion; RV, right ventricular

Parameter	SCM (n=57)	No SCM (n=65)	t/χ²-Value	p-Value
LVEF (%)	44.8 ± 7.5	56.2 ± 6.1	t = 9.00	<0.001
GLS (%)	–13.2 ± 2.9	–17.5 ± 2.6	t = 8.18	<0.001
E/e′ ratio	17.1 ± 4.2	12.5 ± 3.1	t = 6.75	<0.001
TAPSE (mm)	15.2 ± 2.7	18.7 ± 3.0	t = 6.54	<0.001
RV dysfunction, n (%)	22 (38.6%)	7 (10.8%)	χ² = 11.87	<0.001

There were strong correlations between cardiac biomarkers and echocardiographic measures of cardiac function. GLS showed a significant negative correlation with hs-TnI (r = -0.49, p < 0.001), indicating that worsening myocardial strain is associated with higher troponin levels. Likewise, E/e′ ratio had a strong positive correlation with NT-proBNP (r = 0.56, p < 0.001), reinforcing the link between elevated ventricular filling pressures and biomarker levels. In addition, LVEF was negatively correlated with NT-proBNP (r = -0.41, p = 0.002), and TAPSE also showed a moderate inverse correlation with NT-proBNP (r = -0.37, p = 0.006).

**Figure 1:**
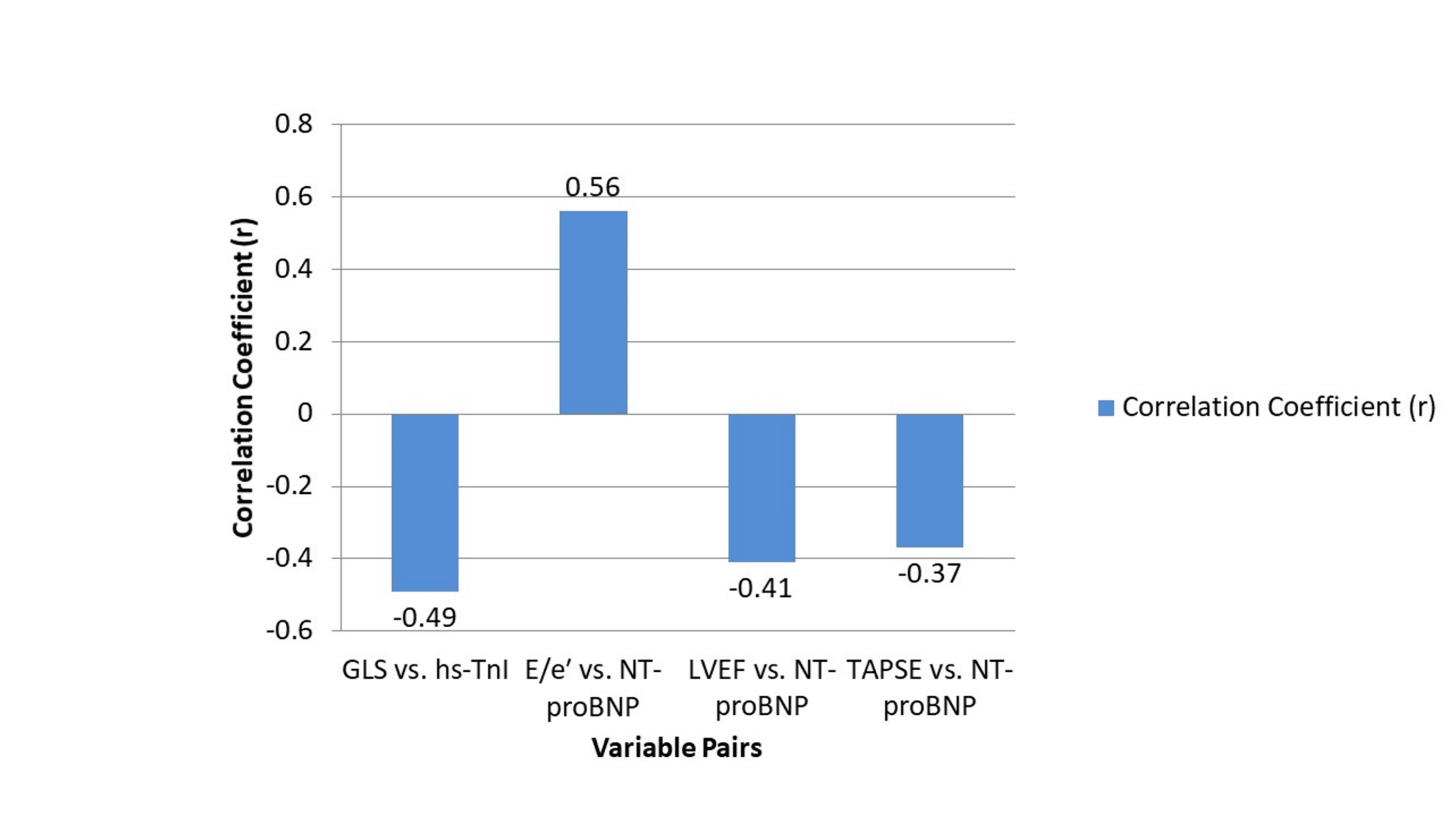
Correlation between echocardiographic and biomarker parameters GLS, global longitudinal strain; hs-TnI, high-sensitivity troponin I; E/e′, ratio of early mitral inflow velocity to early diastolic mitral annular velocity; NT-proBNP, N-terminal pro–B-type natriuretic peptide; LVEF, left ventricular ejection fraction; TAPSE, tricuspid annular plane systolic excursion

To account for potential confounders, multivariable linear regression models were constructed adjusting for renal function (serum creatinine/eGFR) and vasopressor dose as surrogates for afterload. These adjustments attenuated but did not abolish the associations: GLS remained independently associated with hs-TnI (β = -0.32, p = 0.01), while E/e′ retained a significant association with NT-proBNP (β = 0.41, p = 0.004). Although weaker, the associations of LVEF and TAPSE with NT-proBNP remained statistically significant. These results suggest that the observed correlations between biomarkers and echocardiographic parameters reflect true myocardial dysfunction rather than being solely attributable to the pathophysiological state of sepsis. Integrated interpretation of adjusted variables may therefore enhance diagnostic accuracy and treatment decisions in ICU settings (Figure 1).

**Figure 2:**
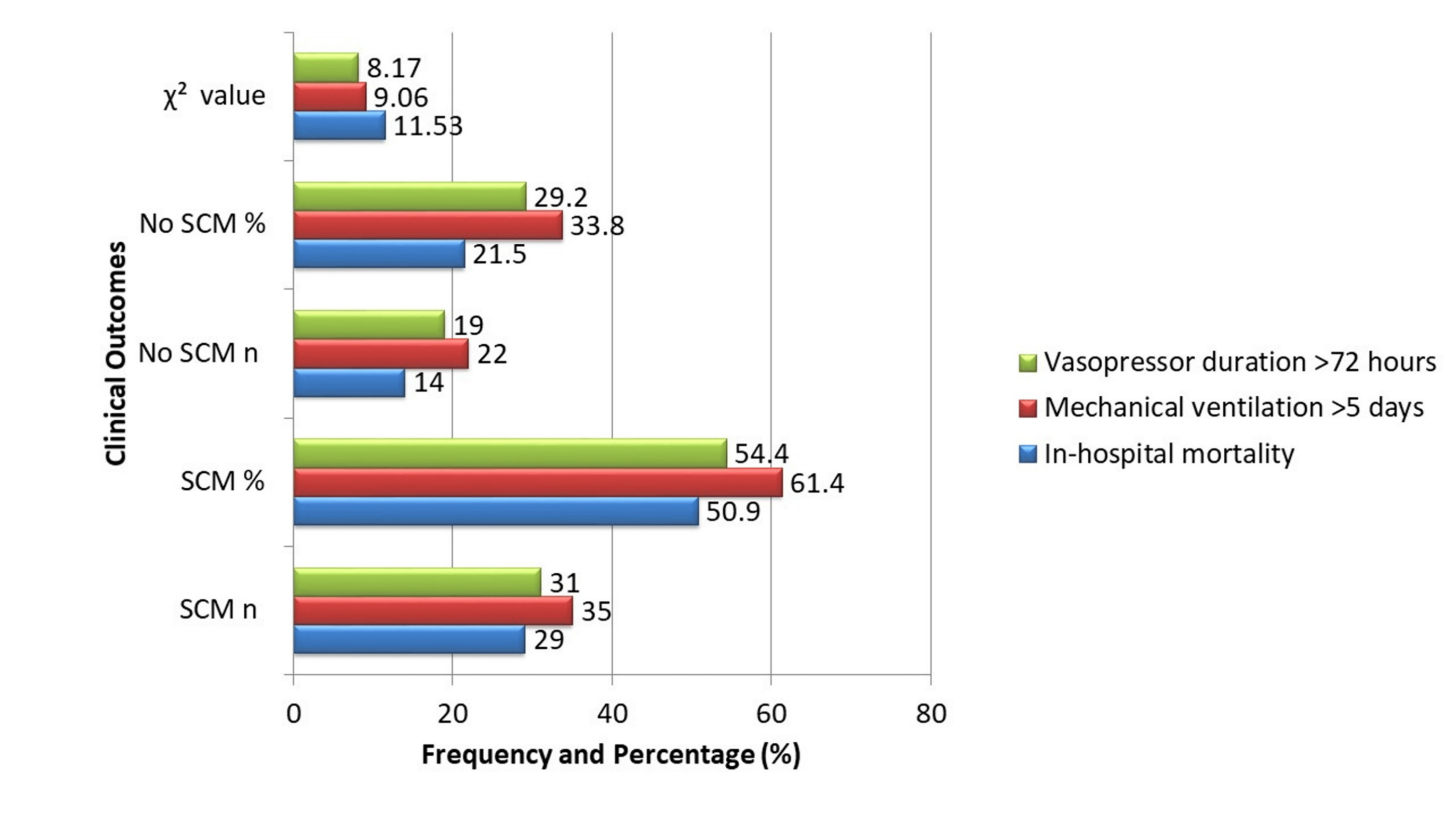
Clinical outcomes in SCM vs. non-SCM patients SCM, septic cardiomyopathy

Patients with SCM experienced significantly worse clinical outcomes compared to those without SCM. In-hospital mortality was observed in 29 (50.9%) of the 57 patients with SCM, compared to 14 (21.5%) of the 65 patients without SCM (χ² = 11.53, p < 0.001). Prolonged mechanical ventilation (>5 days) was required in 35 (61.4%) SCM patients versus 22 (33.8%) non-SCM patients (χ² = 9.06, p = 0.002). Similarly, vasopressor support for more than 72 hours was needed in 31 (54.4%) patients with SCM compared to 19 (29.2%) patients without SCM (χ² = 8.17, p = 0.004). Additionally, the mean ICU length of stay was notably longer in the SCM group, averaging 9.2 ± 3.8 days, compared to 6.5 ± 2.7 days in the non-SCM group (t = 4.42, p < 0.001). These findings highlight the substantial clinical burden and increased resource utilization associated with SCM (Figure 2).

## Discussion

The present study identified SCM in 46.7% of adults admitted with sepsis or septic shock during the 12‑month study period.  SCM was associated with significantly higher illness severity scores, larger cardiac‑biomarker surges, biventricular systolic and diastolic impairment, and a near‑three‑fold rise in in‑hospital mortality.  Notably, LVEF alone underestimated dysfunction; GLS and RV indices exposed additional myocardial injury.  Biomarker trends mirrored imaging: declining troponin‑I and NT‑proBNP by day 3 correlated with improving strain, whereas persistently elevated values heralded adverse outcomes.  Taken together, these findings affirm that a multimodal, serial approach combining echocardiography with biomarkers yields superior risk stratification in septic shock.

The 46.7% prevalence of SCM and the 38.6% rate of RV dysfunction observed in our cohort mirror multicenter systematic review data showing that one‑third to one‑half of septic shock patients develop new LV or RV dysfunction [13]. Like those reports, we found that RV impairment independently tracked with higher APACHE II scores and roughly doubled short‑term mortality, underscoring the prognostic weight of right‑sided mechanics [14]. Our GLS threshold of (-16%) dovetails with cut-offs between -15% and -17% proposed by earlier speckle-tracking studies, which demonstrated that GLS outperforms LVEF in predicting ICU death [15,12]. It is important to note that GLS is influenced by vendor-specific software and age-related variations; reference ranges in healthy populations typically fall between -18% and -22%, with less negative values reflecting subclinical dysfunction. Our cutoff was therefore interpreted in light of these considerations, consistent with published sepsis cohorts.

Peak hs-TnI in this study (~160 ng/L) is consistent with a pooled analysis of >20,000 septic patients, where troponin values 2-3 × the 99th percentile conferred a 1.5- to 2-fold increase in both hospital and long-term mortality [16]. Likewise, our day 1 NT-proBNP concentrations (~5,000 pg/mL) were closely aligned with those reported in acute decompensated heart failure (ADHF) cohorts (median ~5,093 pg/mL), a range that in prior studies was strongly associated with reduced two-year survival and higher recurrent cardiac events [17,18]. This parallel suggests that the biomarker signal we observed in sepsis overlaps with prognostic thresholds established in acute decompensated heart failure, reinforcing its potential utility as a marker of adverse outcomes.

Importantly, because NT-proBNP is strongly influenced by renal clearance and GLS is afterload-sensitive, we performed multivariable adjustment including renal function (serum creatinine/eGFR) and vasopressor dose. The associations between biomarkers and echocardiographic parameters remained significant, though attenuated, confirming that these relationships were not solely driven by the pathophysiological state of sepsis. Integrating adjusted NT-proBNP with diastolic (E/e′) or RV (TAPSE) indices in our analysis therefore provides stronger evidence that biomarker-echocardiography pairings improve risk stratification beyond conventional scores [19].

The in‑hospital mortality of 50.9 % in SCM patients in our study parallels contemporary ICU series and meta‑analyses that place mortality between 40% and 55% when biventricular dysfunction is present [20]. Conversely, the 21.5% mortality in patients without SCM fits the 15-25% range reported for hyperdynamic or preserved function phenotypes [21]. Our finding of a 2.7‑day longer ICU stay and markedly higher rates of prolonged mechanical ventilation corroborates prospective data linking septic myocardial depression to greater resource utilization and ventilator dependence [22].

Limitations and future suggestions

This study has several limitations. Firstly, its single-center design may limit the generalizability of findings beyond this tertiary care setting with its specific patient mix and resource availability. Secondly, although we employed multivariable logistic regression to adjust for age, sex, APACHE II score, vasopressor use, and renal function, residual confounding cannot be fully excluded, and the observational nature of the study precludes causal inference between myocardial indices and clinical outcomes. Thirdly, echocardiographic assessment may have been influenced by vendor-specific software, inter-operator variability, and suboptimal imaging windows in some patients, despite adherence to ASE/EACVI standards and dual-reader validation. Fourthly, while we focused on hs-TnI and NT-proBNP, other biomarkers of myocardial injury and stress (e.g., sST2, GDF-15) were not assessed. Fifthly, treatment was standardized according to Surviving Sepsis Campaign guidelines, but no specific heart failure-directed therapies were applied, which may limit extrapolation to populations where such therapies are incorporated. Finally, follow-up was limited to in-hospital outcomes; post-discharge recovery and long-term functional trajectories could not be evaluated.

Future studies should address these gaps by conducting multicenter, protocolized research to validate GLS and RV-based phenotypes across diverse populations, with standardized adjustment for confounding factors including afterload and renal function. The prognostic value of novel cardiac injury biomarkers in conjunction with strain imaging warrants further exploration. Longer-term follow-up, ideally up to six months, would be valuable in assessing recovery trajectories and identifying late cardiovascular complications. Incorporating advanced imaging techniques, such as 3D echocardiography and cardiac MRI, could further enhance our understanding of SCM pathophysiology.

## Conclusions

This prospective cohort study highlights that SCM is common in critically ill patients with sepsis and is associated with higher illness severity, increased resource utilization, and worse outcomes. Using Sepsis-3-aligned definitions, we found that advanced echocardiographic parameters, particularly GLS and RV indices, together with cardiac biomarkers (hs-TnI and NT-proBNP), provide a more sensitive assessment of myocardial dysfunction than conventional measures such as LVEF alone. Importantly, these associations with mortality and clinical outcomes remained significant after adjustment for confounders including age, severity scores, vasopressor use, and renal function, underscoring the robustness of our findings.

Our results should be interpreted as demonstrating associations rather than causality, and they align with the 2021 Surviving Sepsis Campaign guidance by supporting the role of multimodal assessment in refining risk stratification. Early integration of echocardiographic strain analysis and biomarker monitoring into sepsis care pathways may help identify high-risk patients and guide hemodynamic management, but further multicenter and long-term studies are needed to validate these findings and clarify their impact on patient outcomes.
